# Alternative fate of glyoxylate during acetate and hexadecane metabolism in *Acinetobacter oleivorans* DR1

**DOI:** 10.1038/s41598-019-50852-3

**Published:** 2019-10-07

**Authors:** Chulwoo Park, Bora Shin, Woojun Park

**Affiliations:** Laboratory of Molecular Environmental Microbiology, Department of Environmental Science and Ecological Engineering, Korea University, Seoul, 02841 Republic of Korea

**Keywords:** Soil microbiology, Bacterial genes

## Abstract

The glyoxylate shunt (GS), involving isocitrate lyase (encoded by *aceA*) and malate synthase G (encoded by *glcB*), is known to play important roles under several conditions including oxidative stress, antibiotic defense, or certain carbon source metabolism (acetate and fatty acids). Comparative growth analyses of wild type (WT), *aceA*, and *glcB* null-strains revealed that *aceA*, but not *glcB*, is essential for cells to grow on either acetate (1%) or hexadecane (1%) in *Acinetobacter oleivorans* DR1. Interestingly. the *aceA* knockout strain was able to grow slower in 0.1% acetate than the parent strain. Northern Blot analysis showed that the expression of *aceA* was dependent on the concentration of acetate or H_2_O_2_, while *glcB* was constitutively expressed. Up-regulation of stress response-related genes and down-regulation of main carbon metabolism-participating genes in a Δ*aceA* mutant, compared to that in the parent strain, suggested that an Δ*aceA* mutant is susceptible to acetate toxicity, but grows slowly in 0.1% acetate. However, a Δ*glcB* mutant showed no growth defect in acetate or hexadecane and no susceptibility to H_2_O_2_, suggesting the presence of an alternative pathway to eliminate glyoxylate toxicity. A lactate dehydrogenase (LDH, encoded by a *ldh*) could possibly mediate the conversion from glyoxylate to oxalate based on our RNA-seq profiles. Oxalate production during hexadecane degradation and impaired growth of a Δ*ldh*Δ*glcB* double mutant in both acetate and hexadecane-supplemented media suggested that LDH is a potential detoxifying enzyme for glyoxylate. Our constructed LDH-overexpressing *Escherichia coli* strain also showed an important role of LDH under lactate, acetate, and glyoxylate metabolisms. The LDH-overexpressing *E. coli* strain, but not wild type strain, produced oxalate under glyoxylate condition. In conclusion, the GS is a main player, but alternative glyoxylate pathways exist during acetate and hexadecane metabolism in *A. oleivorans* DR1.

## Introduction

The glyoxylate shunt (GS), a carbon metabolic process from isocitrate to malate via glyoxylate, is a well-known TCA variant during acetate, alkane, and fatty acid metabolism. This GS carbon cycle consists of isocitrate lyase (ICL, encoded by the *aceA* gene) and malate synthase (MS, encoded by either *aceB* or *glcB*). In the β-oxidation cycle, inhibition of FadR by fatty acids inactivates the *aceA* repressor, IclR, inducing expression of the *aceBAK* operon, whose products convert isocitrate to malate through the GS cycle in *Escherichia coli*^1,2^. The GS is also a main carbon flux under several stress conditions, such as oxidative stress, antibiotic stress, cold-/heat-shock, and even desiccation^3–5^. Although the detailed mechanism of the activated GS pathway under conditions of stress is not well studied, it could avoid unnecessary ROS generation by bypassing NADH/FADH production, and respiration, eventually helping cells to survive in harsh conditions^6^.

Due to the absence of a GS cycle in human cells, GS-associated genes and proteins in pathogens could be new targets for antibiotic development^2^. Thus, linkage between GS systems and antibiotics has been extensively studied in several microorganisms. Previously, metabolomic approaches have demonstrated that isoniazid, rifampicin, and streptomycin (antibiotics used for controlling *Mycobacteriumtuberculosis* infection) commonly activate ICL in *M*. *tuberculosis* and an ICL-deficient strain is significantly susceptible to those three antibiotics. However, antioxidants, such as thiourea, could restore the sensitivity to antibiotics, which might imply that the GS pathway is essential for defense against antibiotic action in *M*. *tuberculosis*, and antibiotic-induced oxidative stress could be protected against using an antioxidant in GS-deficient cells^7^. A recent study also showed that the MS is critical for *M*. *tuberculosis* during fatty acid assimilation; furthermore, the elimination of MS in *M*. *tuberculosis* could prevent acute and chronic infections in mice^8^. In another nosocomial pathogen, *Pseudomonas aeruginosa*, both *aceA* and *glcB* were induced under oxidative stress, antibiotics treatment, and iron-limiting conditions^2,9^. When glyoxylate is supplied, *P*. *aeruginosa* is more resistant against tobramycin than in fumarate-supplemented media. Further analysis showed that both reduced respiration rate and proton motive force confer decreased tobramycin uptake and tobramycin resistance^10^.

Although *Acinetobacter baumanii* is a major infectious bacteria and global concerns of its spread have increased due to its multidrug resistance^11^, the GS systems of *Acinetobacter* species remain poorly understood. Reduced persister cell formation appears to be linked to inhibition of ICL, which suggests the importance of ICL in antibiotic resistance (AR) of pathogenic bacteria. However, in a previous study, when colistin or colistin with curcumin was added to *A*. *baumannii*, ICL expression was highly upregulated, and there was limited persister cell formation^12^. There is no general agreement about the role of ICL on persister formation. Living in natural environments also confers many stresses to microorganisms, such as chemical and oxidative stresses, and nutrient deficiencies^13^, which often induces activation of the GS under those harsh conditions. Thus, to gain a better understanding, further research of the GS is required for not only pathogens, but also environmental microorganisms.

Previously, our lab has shown that GS-participating genes in a soil-borne bacterium, *A*. *oleivorans* DR1, are upregulated upon exposure to ampicillin, paraquat (PQ), phenazine methosulfate (PMS), hexadecane (Hex), and triacontane (TRI)-treated condition^14–18^. Examination of an *aceA*-deficient strain in triaconatane (C30 alkane)-containing minimal salt basal (MSB) media showed retarded growth with a long lag phase in contrast to that observed for the parent strain, and the susceptibility of the ICL-lacking mutant to H_2_O_2_ was considerably increased^17^. Thus, alkane or ROS-generating substances can induce carbon-metabolic shift to GS in *A*. *oleivorans* DR1. However, detailed mechanisms are still in its early stage. In this study, we focused on analyzing unpredicted growth of GS-deficient DR1 cells under 0.1% acetate and Hex. Our data suggested that low toxicity of 0.1% acetate and low solubility of Hex enable ICL-lacking DR1 cells to grow using an unknown alternative pathway and MS-lacking cells produce oxalate under the same conditions for detoxifying accumulated glyoxylate.

## Results and Discussion

### Comparative growth of WT and GS null-strains in the presence of acetate and Hex

Previously, it was shown that GS-related genes in DR1 are highly upregulated when cells degrade Hex and TRI^16,17^. The Δ*aceA* mutant grew at a slower rate than the WT in Hex-, hexadecenoic acid-, and TRI- supplemented media, and no growth was observed in 1% NaAc-amended media^17^. However, the susceptibility of the *aceA* mutant under H_2_O_2_ treatment and the induction of *aceA* expression in the WT as a result of increased H_2_O_2_ concentration indicates that *aceA* is also critical for survival of the DR1 strain under conditions of oxidative stress. On the other hand, deletion of the *glcB* gene did not increase the sensitivity to H_2_O_2_ (Fig. S1). This result led to the assumption that an alternative GS pathway is present.

**Figure 1:**
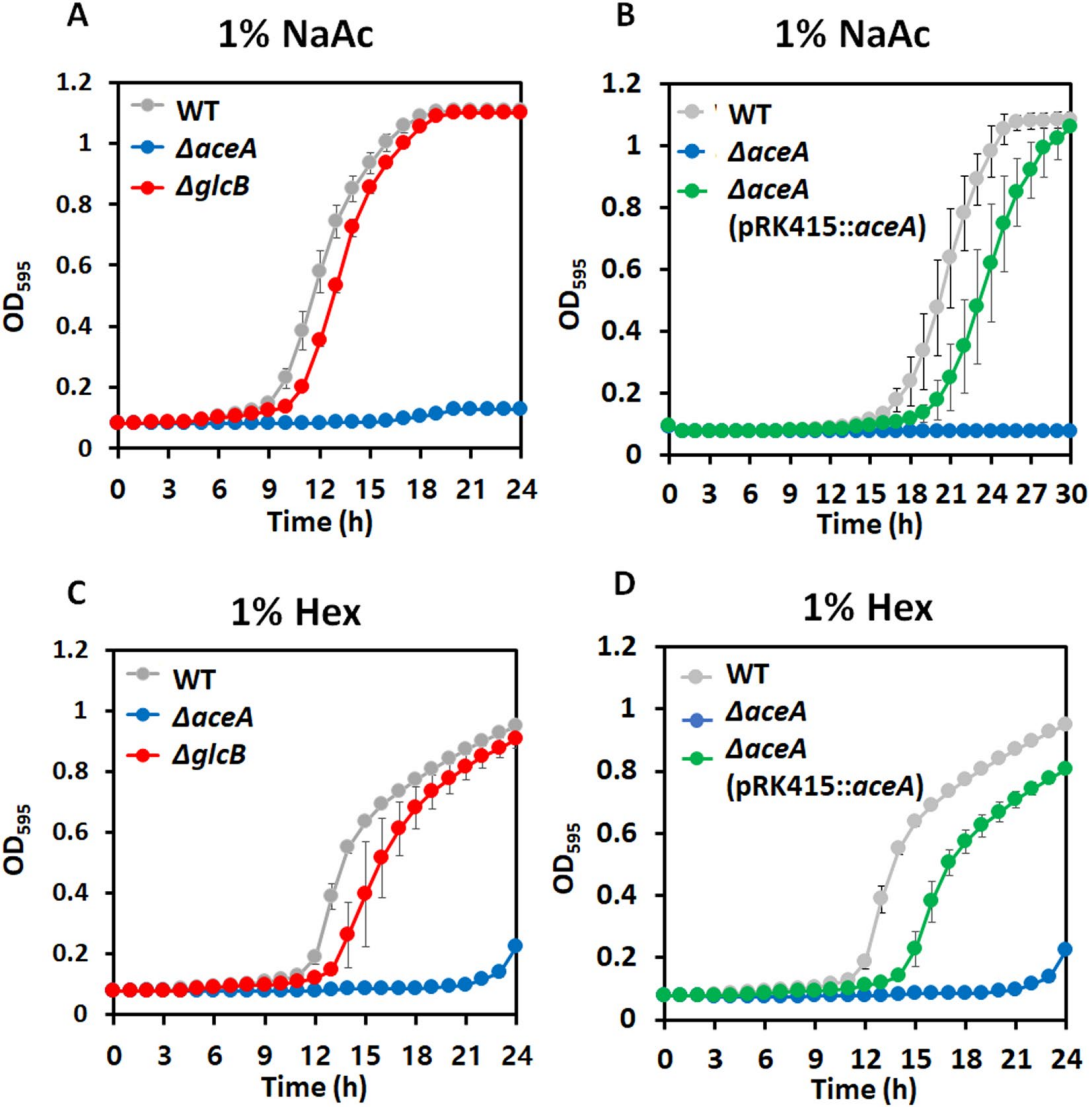
Comparative growth of wild type (gray), Δ*aceA* (blue), and Δ*glcB* (red) strains in the presence of 1% NaAc and 1% Hex. (**A**) Wild type and Δ*glcB* mutant strains grew, but Δ*aceA* mutant did not in 1% NaAc-added MSB media. (**B**) Complementation of the *aceA* mutant (green) was restored in the same condition as Panel A. (**C**) The same growth patterns were observed for the three strains in 1% NaAc-supplemented MSB as in 1% Hex-supplemented media. (**D**) The growth defect of the *aceA* mutant was also restored in the presence of 1% Hex by complementation. All data show the average of three replicates, and the error bar indicates the standard deviation.

Growth assays were conducted under 1% sodium acetate (NaAc) and Hex. Surprisingly, the WT strain and *glcB* null-mutant strains of *A. oleivorans* DR1 grew normally with similar growth rates under both conditions (Fig. 1A,C). On the other hand, growth of *aceA*-lacking mutant was not observed under the same conditions (Fig. 1A,C). Due to extreme toxicity of glyoxylate generated during NaAc and Hex metabolism^3,8^, an alternative pathway of glyoxylate must exist for survival of the *glcB* null-mutant under such conditions. Growth inhibition of Δ*aceA* cells was restored by *aceA* complementation under 1% NaAc and Hex (Fig. 1B,D). From the above, it is apparent that ICL, but not MS, is essential, and an alternative pathway of glyoxylate in MS-lacking cells exists during 1% NaAc and Hex metabolism.

*Escherichia coli*, *aceA* and *aceB*, encoding ICL and MS, are clustered as an operon, so that they are expressed and regulated simultaneously^19^. However, many *aceA* and *glcB* (or *aceB*) genes had dispersed from one another in the genomes of many bacteria^9^, indicating that divergently evolved ICL and MS might participate in the same process accidentally. In this context, it is possible that other enzymes could play a role in glyoxylate detoxification generated by ICL.

### Evaluation of acetate toxicity in GS-deficient mutants

**Figure 2:**
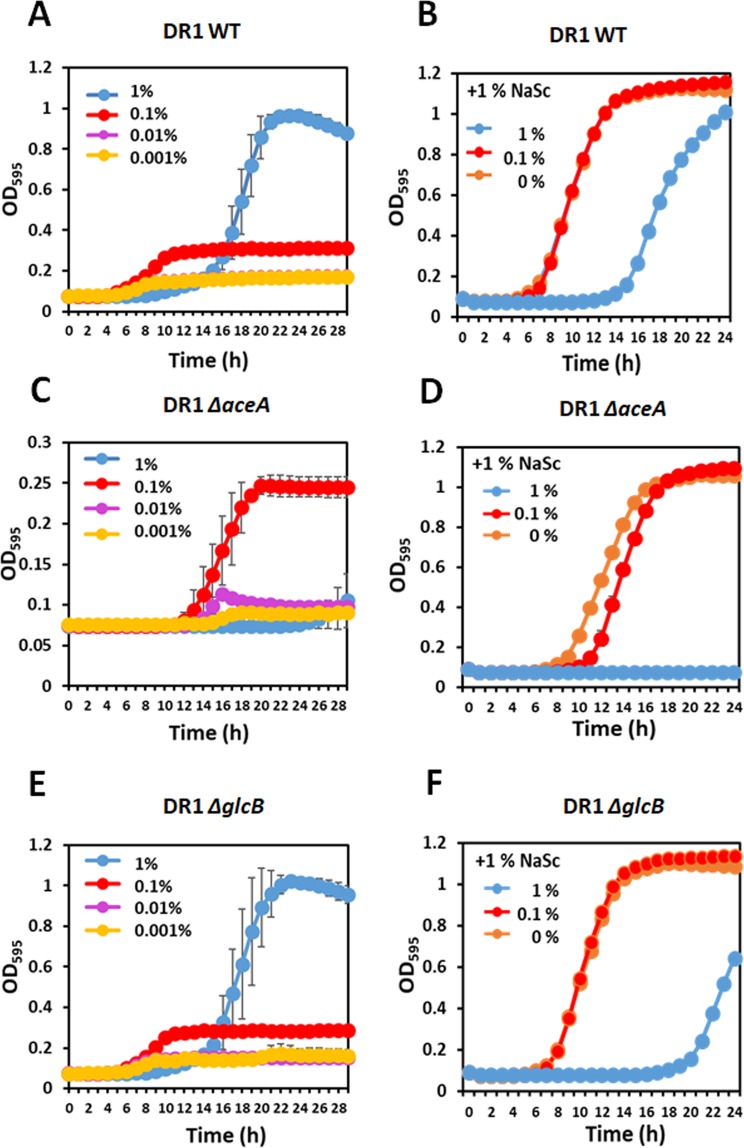
The growth of wild type, Δ*aceA*, and Δ*glcB* strains in the various concentrations of NaAc. (**A,C,E**) DR1 wild type, Δ*aceA*, and Δ*glcB* mutants were inoculated in 0.001 (yellow), 0.01 (purple), 0.1 (red), and 1% (blue) NaAc-supplemented MSB media, the cultures were incubated at 30 °C, and optical density was observed at 595 nm (OD_595_). (**B,D,E**) DR1 wild type, Δ*aceA*, and Δ*glcB* strains were cultured when 0 (orange), 0.1 (red), and 1 (blue) NaAc was added to 1% NaSc-supplemented MSB media. The graphs show the average of three replicates, and the error bars indicate the standard deviation.

Similar growth patterns of WT and Δ*glcB* mutant strains were observed; the highest growth rate and the longest lag phase were observed in 1% NaAc among the surveyed conditions of 0.01–1% NaAc-supplemented media (Fig. 2A,C). Surprisingly, growth of the *aceA* knock-out strain has been observed under 0.1% NaAc and a final OD_600_ could reach 0.25 (Fig. 2B). We hypothesized that the essentiality of the *aceA* gene under acetate metabolism in many bacteria requires reconfirmation because all experimental reports used high concentrations of acetate and fatty acids^9,20,21^. The sensitivity of the *aceA* mutant in 1% NaAc would be due to the toxicity of acetate because the *aceA*-null strain also showed high susceptibility to acetate even when another carbon source, sodium succinate (NaSc), which activates the TCA cycle, is present (Fig. 2D). The WT and Δ*glcB* mutant strains grew in NaAc and NaSc-mixed media (0.1% NaAc and 1% NaSc), however, retarded growth of both strains was shown in the presence of 1% NaAc plus 1% NaSc (Fig. 2D,F). Furthermore, the *aceA* mutant could not grow in 1%-NaAc and NaSc supplemented media, and growth was slightly delayed in 0.1% NaAc and 1% NaSc media compared to that in only 1% NaSc-supplemented media (Fig. 2E).Retarded growth of the WT and Δ*glcB* strains and inhibited growth of the Δ*aceA* mutant were observed in the presence of 1% NaAc with or without NaSc (Fig. 2A,B,E,F), indicating that 1% NaAc is sufficiently toxic to the DR1 strain.

**Figure 3:**
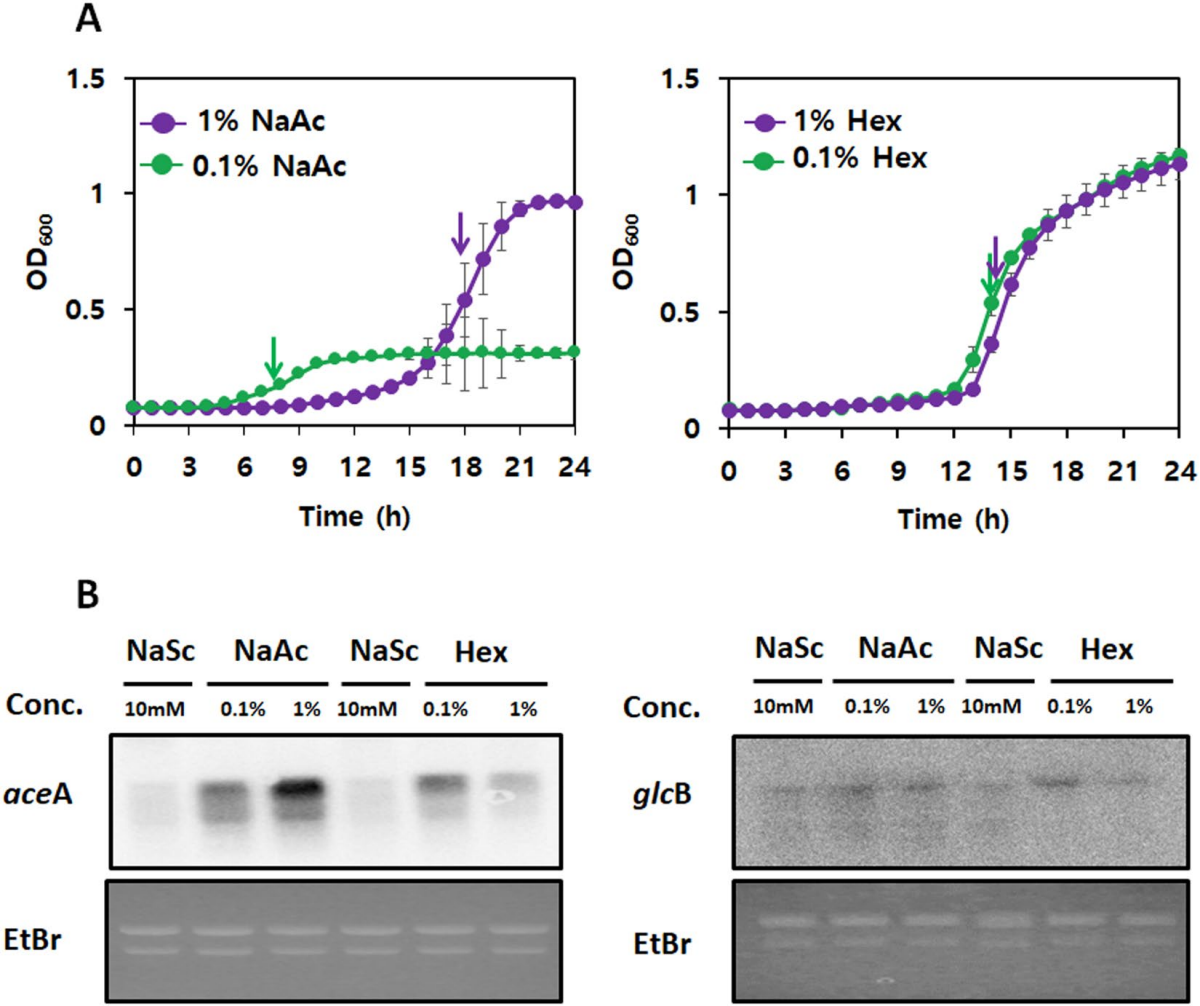
Growth and expression of wild type strain in 0.1 and 1% NaAc and Hex. (**A**) Growth of DR1 wild type strain in 0.1% (green) and 1% (purple) NaAc and Hex was monitored over 24 hr by measuring the optical density at 600 nm wave-length. Arrows indicate the point at which total RNA was extracted to perform northern blot analysis. Three replicates were conducted, and the error bar indicates the standard deviation. (**B**) Northern blotting was conducted to evaluate gene expression of *aceA* and *glcB* when DR1 was grown during the mid-exponential phase in 10 mM NaSc (OD_600_~0.5), 0.1- (OD_600_~0.15)and 1% NaAc (OD_600_~0.5), and 0.1- and 1% Hex (OD_600_~0.5) -supplemented MSB media.The images of gel and blot between *aceA* and *glcB*were cropped from different gels, and full-length blots/gels are presented in Supplementary Fig. S2. To clearify the expression of *glcB* gene, high-contrast images of gel and blot were shown (Supplementary Fig. S2).

Acetate toxicity to cells has mainly two outcomes: 1. release of protons in the cytoplasm, and 2. intercalation of undissociated acetate into the lipid bilayer at low external pH^22^. A previous study has shown that 1% NaAc did not lower external pH^17^, thus it is expected that the growth deterioration of DR1 cells is due to intracellular acidification in high concentration of acetate (>1% NaAc). Interestingly, the Δ*aceA* mutant could not grow in the presence of 1% NaAc but showed slight growth in 0.1% NaAc-supplemented media (Fig. 2C,D), implying that a low concentration of NaAc (0.1%) could be metabolized without ICL, but not as efficient as in the GS pathway. Unlike growth in the presence of NaAc, the growth rate and the expression of *aceA* and *glcB* were not significantly different between 1% and 0.1% Hex (Fig. 3). In fact, several obstacles are present when DR1 metabolizes Hex; the Hex solubility in media, the diffusion rate of Hex through the cell membrane, and the activation rate of inert Hex. Thus, the observation of no differences in growth rate or gene expression on 1% and 0.1% Hex are probably due to the above limited factors.

### Expression of GS-participating genes and growth under high and low concentrations of NaAc and Hex

To confirm the importance of the GS during acetate and Hex metabolism, the expression of *aceA* and *glcB* was quantified using a Northern blot assay. Previously, many studies have reported that oxidative stress induces activation of the GS pathway in *Mycobacterium tuberculosis*, *Pseudomonas aeruginosa*, as well as *A*. *oleivorans* DR1^3,7,17^. It is generally agreed that the GS pathway helps cells avoid unnecessary NADH production, which prevents excess ROS generation from unbalanced oxygen consumption during stress conditions^20^. The *aceA*-deficient strain is highly sensitive to H_2_O_2_ compared to the WT or *glcB*-knockout mutant (Fig. S1A–C). The expression of *aceA* and *glcB* was examined under oxidative stress conditions (0-, 0.1-, 0.2-, 0.5-, and 1 mM H_2_O_2_). The expression of *aceA* was considerably increased only when a high concentration of H_2_O_2_ (1 mM) was added (Fig. S1D). Low concentrations of H_2_O_2_ might be quickly eliminated by various catalases present in the genome of DR1 cells^23^. On the other hand, *glcB* showed constitutive low-level expression (Fig. S1E). The expression of *aceA* was also significantly increased in the presence of NaAc and Hex compared to that observed in the presence of NaSc (Fig. 3A). Interestingly, *aceA* was expressed in a NaAc concentration-dependent manner, but the expression level under Hex was not increased even at a high concentration because of the low solubility of Hex. Constitutive expression of *glcB* gene was observed on 0.1 and 1% NaAc, however, expression was decreased in 1% Hex compared to that in 0.1% Hex (Fig. 3A). A relatively longer lag time, but higher optical density values in 1% NaAc (OD_600_~1.0) compared to that in 0.1% NaAc (OD_600_~0.3) were observed at the stationary phase, which also offers an insight into acetate toxicity at high concentration. However, similar growth rates and optical density values were observed in 0.1 and 1% Hex-supplemented media (OD_600_~1.1) (Fig. 3B). Our expression and deletion analyses suggested that the *aceA* product plays significant protective roles under conditions of oxidative stress by operating the GS and an unrecognized pathway might be present for replacing the role of the *glcB* product.

### Transcriptome analysis of *aceA* mutant during acetate metabolism

To analyze the mechanism for detoxifying acetate in GS-deficient strains, transcriptomic analysis was performed. Previous RNA-seq data of DR1 under succinate-supplemented media were retrieved^17^ and compared with transcriptome data of WT and *aceA* mutant strains when they were grown in 0.1% NaAc-supplemented media in this study. The information of analyzed RNA-seq data is supplied in Table S1. Compared to that of the WT strain, 272 and 1,096 genes were up-, and down-regulated, respectively, in the *aceA* mutant. In addition, 63.8% of all genes showed no change at the transcriptional level, implying that no significant metabolic alterations would occur (Fig. S3A). Six of the ten most upregulated genes encoded hypothetical proteins, and the remaining genes were annotated as a bacterial RNaseP, ferredoxin, a murain hydrolase, and an aldehyde dehydrogenase (Fig. S3B). The highest upregulated gene, encoding a hypothetical protein, showed only a 2.3-fold increase in the aceA mutant compared to that in the control (Fig. S3B).

In WT cells, downstream genes (succinate to oxaloacetate) of the TCA cycle (*sucCD*, *sdhABCD*, *fumBC*, *mqo*, and *mdh*) were down-regulated during acetate metabolism, compared to that under succinate-supplemented conditions (Fig. S4). Upstream genes (citrate to succinyl CoA) of the TCA cycle (*gltA*, *acnB*, *icd*, and *sucAB*) including GS-participating genes (*aceA*, and *glcB*) in WT strain were upregulated in the NaAc-supplemented media compared to that in NaSc-supplemented media, confirming that acetate metabolism occurs actively through the GS and upstream of TCA cycles (Fig. S4). Furthermore, *aarC*, encoding a succinyl-CoA:acetate CoA transferase, was highly upregulated in the presence of NaAc (WT RPKM_NaSc_, 409.2; WT RPKM_NaAc_, 2011.1; Δ*aceA*RPKM_NaAc_, 1701.8, Fig. S5A). These results indicate that acetate is also possibly metabolized by succinyl-CoA:acetate CoA transferase generating succinate and succinyl CoA in *A*. *oleivorans* DR1 (Fig. S5).

Notably, *fadB* and *fadA*, encoding an enoyl-CoA hydratase/3-hydroxybutyryl-CoA epimerase/a 3-hydroxyacyl-CoA dehydrogenase and 3-ketoacyl-CoA thiolase, respectively, showed slightly higher expression levels in the aceA-null mutant compared to that of the WT. In addition, poly-3-hydroxybutyrate (PHB) synthesis participating genes, *phaA* (Acetyl-CoA acetyltransferase) and *phaB* (Acetoacetyl CoA reductase), were also slightly upregulated in the *aceA*-null strain (Fig. S5B). However, expression of *phaC* (PHB synthase) and *phaZ* (PHB depolymerase) was not significantly increased in the *aceA*-lacking strain (*phaC* 1.0-fold; *phaZ* 0.9-fold). Thus, acetyl CoA might be forced to 3-hydroxy butyryl-CoA via acetoacetyl-CoA due to a retarded consumption rate for acetyl-CoA.

Cells lacking the GS need to maintain the levels of NADH and ATP by operating other cellular metabolism pathways. Iron is an essential element for enzymes participating in the TCA cycle and electron transport chain (ETC)^24^. It has been shown that iron limitation induces the GS cycle and acetate-triggered GS operating cells also have lower intracellular iron content^9^. In the Δ*aceA* mutant, genes related to iron transporters (*fec*, and *feoB*), iron regulatory protein (*fur*), iron-binding protein (*bfr*), ferredoxin (*fdx*), and glutaredoxin (*grx*) were upregulated (Table S2). Furthermore, the expression levels of genes involved in the ETC (*cyoB*, cytochrome O ubiquinol oxidase; *nuoI*, NADH-quinone oxidoreductase subunit I; and *etfD*, probable electron transfer flavoprotein-ubiquinone oxidoreductase) and ATP synthase subunits (*atpE*, *atpB*, *atpC*, and *atpA*1) were slightly increased with high RPKM (>1,000) in the *aceA* mutant (Table S2). Taken together, increased expression level of genes related to iron uptake, respiration, and ATP synthesis occur in the *aceA* mutant because of compensation for GS malfunction.

RNA-seq data implied that the *aceA*-null mutant is under severe stresses compared to the WT strain. Three cold shock protein-coding genes (*cspA*, *cspE*, and *cspG*) were upregulated when the *aceA* was deleted. Notably, *cspA*, and *cspE* are considerably upregulated in the knockout strain (1.5-, and 1.4-fold, respectively) with high RPKM (>1,000). In addition, DNA repair proteins (encoded by *recO*, and *uvrC*), DNA/RNA helicases (encoded by *hrpA*, and *ruvX*), and oxidative stress response proteins (encoded by *ahpF*, and *katG*) were expressed to a greater extent in the *aceA* knockout mutant, indicating that the lack of the GS confers acetate susceptibility to the mutant.RNA-seq profiling showed that activated Pta-AckA (reversible pathway from acetate to acetyl CoA) and the GS are main pathways during acetate metabolism (Fig. S4). Furthermore, the high expression of *aarC* in NaAc implies an alternative contributing factor for acetyl-CoA utilization in the presence of acetate (Fig. S5A). It was reported that *aarC* confers acetate resistance and assimilation to *Acetobacter aceti*, a GS-lacking bacterium^25^. *A*. *baumanii* also possesses an *aarC* gene and the function of Succinyl-CoA:acetate CoA transferase was examined^26^. Therefore, *aarC* could be a candidate for acetate metabolism when glyoxylate is inactivated in *A*. *oleivorans*. The expression level of *aarC* in the Δ*aceA* mutant strain is slightly lower than that of the WT in the presence of NaAc, resulting in the retarded growth of the *aceA*-null mutant compared to that of the WT strain (Figs 2 and S5A). Furthermore, the Δ*aceA* mutant may synthesize acetoacetyl CoA and 3-hydroxybutyryl CoA to avoid acetyl CoA accumulation (Fig. S5B). Upregulated genes for alkane degradation are ambiguous, the genes maybe induced due to the acetate accumulation in the *aceA* mutant^27^. Previous studies have revealed that respiration- and proton motive force-related genes were downregulated in *P. aeruginosa* when glyoxylate is a sole carbon source, because it uses the GS cycle rather than the TCA cycle^10^. Thus, blocking of the GS by elimination of the *aceA* gene may cause cells to sustain respiration and proton motive force. Higher expression of cytochrome oxidase, Fe-S cluster proteins, and iron transporter was observed in the *aceA* mutant compared to that of the wild type. Marine heterotrophic bacteria has evolved to survive in iron-limited conditions by activating the GS and reducing iron consumption rate (reducing the expression of Fe-S cluster participating in the TCA cycle and ETC)^9,24^.

Expression of genes for motility, such as *cheY* (Chemotaxis protein), *pilY* (Twitching motility protein), *pilW* (Pilus assembly protein), *pilF* (Pilus assembly protein), and *pilS* (Sensor protein), was increased in the *aceA*-null strain compared to that in the WT strain, suggesting that lack of GS might be responsible for active motility. Although the relationship between motility and GS is unclear, a recent study demonstrated that cold shock protein C (CspC) positively regulates the expression of GS (*aceA*) and motility (*fljNLK*, *pilA*, and *cheW*) related genes^28^. Thus, it could be hypothesized that abundant Csps within the *aceA* mutant cell confers increased expression of motility-related genes directly or indirectly.

### Alternative glyoxylate detoxification by lactate dehydrogenase (LDH)

**Figure 4:**
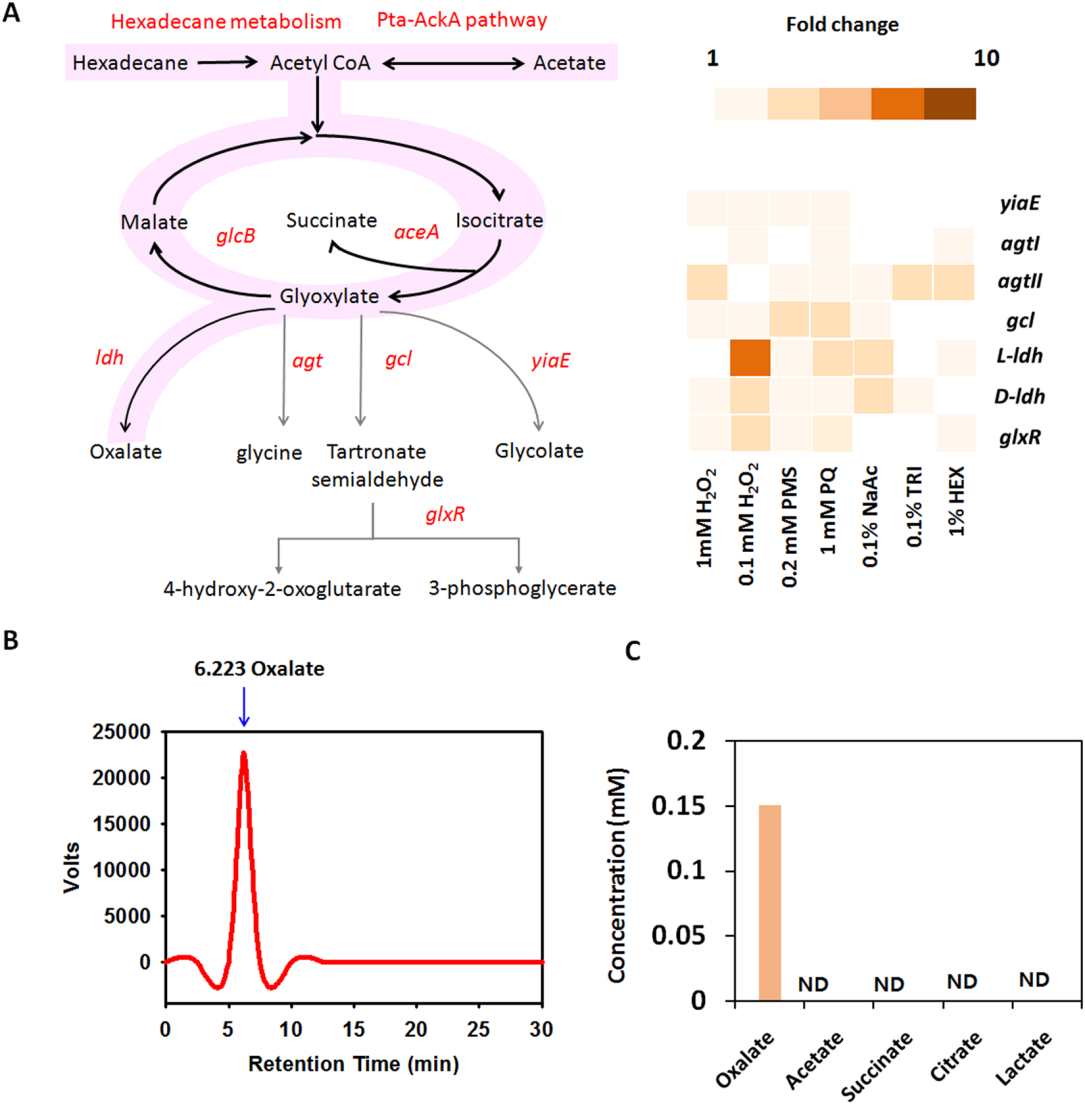
Candidates for alternative GS pathways of glyoxylate detoxification. (**A**) The expected alternative pathways in *A. oleivorans* DR1 and the expression of candidates for participating genes under several oxidative stress conditions. Intensity of color represents the expression levels of the genes. The following genes are candidates: glyoxylate/hydroxypyruvate reductase B (*yiaE*), glyoxylate carboligase (*gcl*) lactate dehydrogenase (*ldh*), alanine-glyoxylate aminotransferase (*agt*), and tartronate-semialdehyde reductase (*glx*). The treated reagents conferring stress to cells were peroxide (H_2_O_2_, 0.1-, 1-mM), phenazine methosulfate (PMS, 0.2 mM), paraquat (PQ, 1 mM), sodium acetate (NaAc, 0.1%), triaconatane (TRI, 0.1%), and hexadecane (Hex, 1%). Thick black dash lines with pink-background indicate a typical main pathway during Hex, and NaAc metabolism, on the other hand, a thin black dash line with pink-background represents a proposed alternative pathway in this study. Gray arrows are pathways which were previously described in several literatures^33,35,36^, but not evaluated in this organism. (**B**) The chromatogram of high-performance liquid chromatography (HPLC). X-axis and Y-axis indicate retention time and signal intensity, respectively. The peak of oxalic acid was detected at 6.223 min. (**C**) Organic acid profiling of DR1 grown in 1% Hex-supplemented MSB media. The concentration of organic acids in the supernatant of DR1. The concentration (μg/g) of organic acid is shown in the Y-axis. ND indicates ‘not detected’.

The phenotypic differences between WT and *glcB* knockout strains were not noticeable, although the GS is not operating in the *glcB* mutant under NaAc- or Hex-supplemented media or under conditions of oxidative stress (Figs 1 and S1). Thus, we assumed that DR1 possesses an alternative pathway for detoxifying accumulated glyoxylate during NaAc- or Hex metabolism. Using BlastP, possible candidate enzymes for glyoxylate detoxification were searched, and seven genes which are homologous to hydroxypyruvate/glyoxylate reductase, glyoxylate carboligase, alanine glyoxylate transaminase, lactate dehydrogenase (LDH), and tartronic semialdehyde reductase were found (Table S3, Fig. 4A). Furthermore, previous RNA-seq data were retrieved, and the gene expression of candidates under 0.1 and 1 mM H_2_O_2_, 0.2 mM PMS, 1 mM PQ, 0.1% NaAc, 0.1% TRI, and 1% Hex treatment was investigated^15–17^. Upregulation of *L-ldh* and *D-ldh* genes were observed in most of these conditions (1 mM H_2_O_2_, PMS, PQ, NaAc, and HEX = 5.9-, 1.1-, 2.4-, 2.2-, 0.9-, and 1.1-fold changes for *L-ldh*; 2.9-, 1.2-, 1.4-, 2.0-, 1.0-, and 0.9-fold changes for *D-ldh*) (Fig. 4A). Many previous studies have suggested that glyoxylate is a substrate for a LDH in human erythrocytes, human plasma, rabbit muscle, rat liver, spinach, and pig heart, resulting in the production of oxalate^29–31^. LDH might have broad substate specificity.

**Figure 5:**
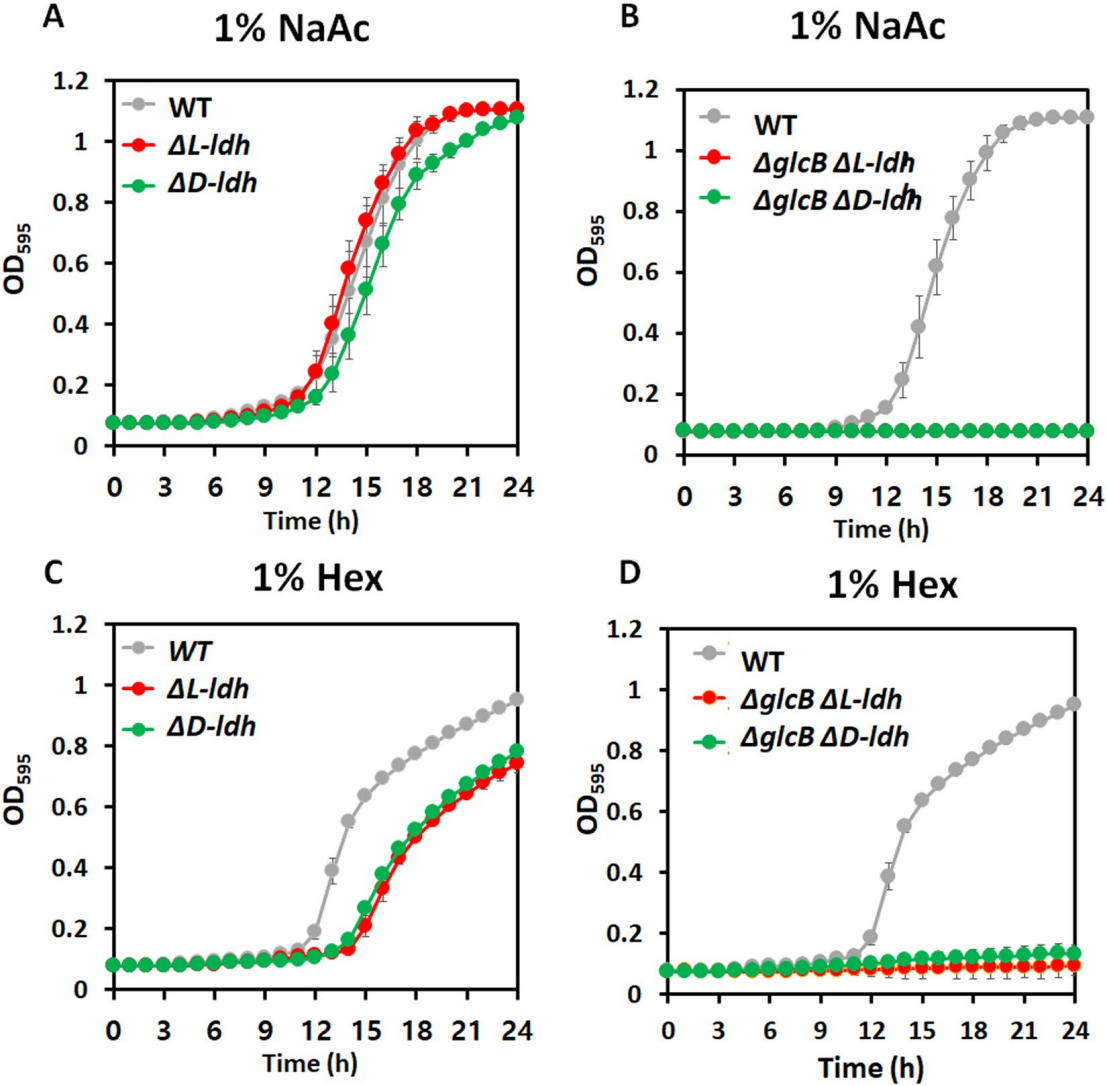
Comparative growth of wild type, Δ*ldh*, and Δ*glcB*Δ*ldh* strains. The growth of: (**A**) wildtype (gray), single Δ*L-ldh* (red), and Δ*D-ldh* (green) strains and (**B**) wild type (gray), double Δ*glcB*Δ*L-ldh* (red), and Δ*glcB*Δ*D-ldh* (green) strains in 1% NaAc-supplemented media over 24 h. Growth of: (**C**) wild-type (gray), single Δ*L-ldh* (red), and Δ*D-ldh*(green) strains; and (**D**) wild type (gray), double Δ*glcB*Δ*L-ldh* (red), and Δ*glcB*Δ*D-ldh* (green) strains in the presence of 1% Hex. Growth was monitored by the measurement of optical density at 595 nm (OD_595_). All data show the average of three replicates, and the error bar indicates the standard deviation.

**Figure 6:**
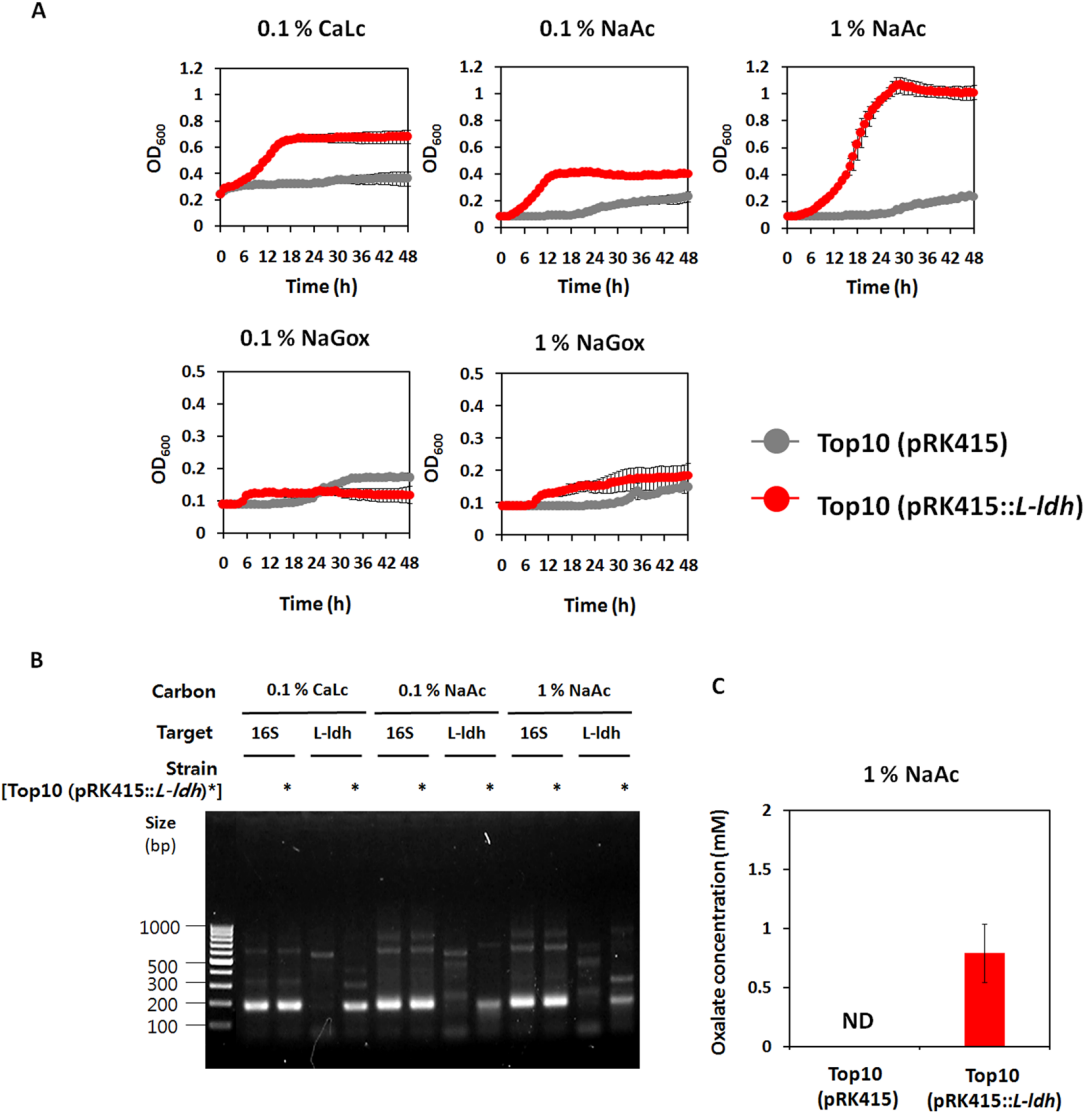
Examination of LDH expression in *E.coli* Top10 during acetate metabolism. (**A**) The growth curves of *E.coli* Top 10 (pRK415; gray line) and *E.coli* Top 10 (pRK415::*L-ldh*; Red line) in 0.1% calcium lactate (CaLc), 0.1-, and 1% sodium acetate (NaAc)-supplemented media during 48 h. Growth was monitored by the measurement of optical density at 600 nm (OD_600_). All data show the average of three replicates, and the error bar indicates the standard deviation. (**B**) The expression profile of 16S rRNA (193 bp) and *L-ldh* (191 bp) in Top 10 (pRK415) and Top 10 (pRK415::*L-ldh*). The expression of Top 10 (pRK415::*L-ldh*) is highlighted by asterisk. After PCR from 1/10-diluted samples, 5 μL of samples were loaded to ethidium bromide-stained gel. (**C**) Intracellular oxalate concentration (mM) of Top 10 (pRK415) and Top 10 (pRK415::*L-ldh*) during 1% NaAc metabolism. ND indicates ‘not detected’.

In addition, the expression of genes encoding a lactate dehydrogenase (*L-ldh*, and *D-ldh*) is relatively higher among potential enzymes for glyoxylate as a substrate during NaAc-, and alkane metabolism (Fig. 4A). Thus, we hypothesized that a LDH is one of the promising enzymes for glyoxylate detoxification in *A*. *oleivorans* DR1 although no studies have elucidated the activity of LDH for glyoxylate as a substrate in bacteria. Interestingly, only oxalate (0.15 mM) was detected in the supernatant of Hex-supplemented media, implying that oxalate can be produced during Hex metabolism, which is one of the GS-activating conditions in the DR1 strain (Fig. 4B,C).Single *ldh* knockout strains (Δ*L-ldh* and Δ*D-ldh*) could grow (Fig. 5A,C), but the growth of *ldh*-, *glcB* double knockout strains (Δ*L-ldh*Δ*glcB* and Δ*D-ldh*Δ*glcB*) was completely impaired in both NaAc and Hex-supplemented media, implying that the *ldh* gene product compensates the lack of the *glcB* gene during acetate and Hex metabolism by detoxifying glyoxylate (Fig. 5B,D). To conduct further analyses of LDH, we cloned the *L-ldh* gene of *A*. *oleivorans* DR1 into pRK415 vector and generated *Escherichia coli* Top10 expressing *L-ldh* gene [hereinafter referred to as Top 10 (pRK415::*L-ldh*)]. Because LDH is known to mediate the reaction from lactate to pyruvate, vice versa. Growth of Top 10 (pRK415::*L-ldh*) under lactate, acetate or glyoxylate was monitored. Control strain having only the vector could not grow well under all tested conditions, but LDH from *A*. *oleivorans* DR1 cells boosted the growth of Top 10 (pRK415::*L-ldh*) under the same conditions (Fig. 6A). Poor growth of control strain under acetate or glyoxylate might be due to their toxicities to cells, which could not be observed in the presence of LDH (Fig. 6A). Reverse transcription-polymerase chain reaction (RT-PCR) confirmed that *L-ldh* gene in Top10 (pRK415::*L-ldh*) strain was expressed during lactate and acetate metabolisms (Fig. 6B). Suprisingly, Top10 (pRK415::*L-ldh*) strain, but not Top 10 (pRK415), produce oxalate (0.79 mM) duering acetate metabolisms. These data supports the possible role of LDH in the alternative fate of glyoxylate during acetate metabolism.

In summary, we demonstrated that the *aceA* mutant strain could grow at a low concentration of acetate and suggested that succinyl-CoA:acetate CoA acetyl transferase is a candidate for acetate metabolism. Furthermore, it was concluded that glyoxylate detoxification is conducted by a lactate dehydrogenase in the *glcB* mutant. Although the GS pathway is a main carbon flux in DR1, an alternative pathway via lactate dehydrogenase also exists to enable survival under several dynamic environments.

## Methods

### Bacterial strains, chemical reagents, and culture conditions

All growth tests for *A*. *oleivorans* DR1 were conducted at 30 °C in MSB media^14^ with medium- intensity agitation for 100 sec every 1 h. Seed culture of DR1 strain was grown at 30 °C in nutrient broth (NB) medium. Growth was monitored by measuring the optical density at 595 nm (OD_595_) of cultures using a tecan microplate reader (Surnrise, Switzerland). The complete genome sequence of *A*. *oleivorans* DR1 can be assessed in GenBank (accession no. CP002080). *A*. *oleivorans* DR1 was deposited in the Korea Collection for Type Cultures (KCTC 23045) and the Japan Collection of Microorganisms (JCM 16667). Hydrogen peroxide (H_2_O_2_) and pyruvate were purchased from Junsei and Bioshop, respectively. All other following chemicals were purchased from Sigma-aldrich (USA); sodium acetate, hexadecane, sodium glyoxylate, sodium oxalate, calcium lactate.

### Construction of mutants

*aceA* knockout and complemented strains were produced as described previously^17^. To construct the cloning vector for knockout, the internal region of the *glcB*, *L-ldh*, *D-ldh* genes was amplified using PCR from genomic DNA (Table S4). A *glcB* PCR product and pVIK 112 plasmid were digested using KpnI and SmaI restriction enzymes, fragments of *L-ldh* and *D-ldh*, and pEX18Gm were treated with KpnI and EcoRI restriction enzymes. Each fragment was subsequently cloned into the pVIK 112 plasmid for *glcB* genes and the pEX18Gm plasmid for *L-ldh* and *D-ldh* genes via ligation. The constructed plasmids were then transformed into *E. coli* S17-1λ pir strain, extracted from *E. coli*, and transformed into *A. oleivorans* DR1. Knockout mutants were screened on nutrient agar (NA) containing antibiotics (50 μg/ml kanamycin, or 15 μg/ml gentamycin). To produce ldh and glcB double KO mutants, vectors were constructed (pEX18Gm::*ldh*) as above, introduced into *glcB* single KO strain, and then the double KO mutant was selected on NB agar containing 50 μg/ml kanamycin and 15 μg/ml gentamycin.

### RNA extraction, library construction, and sequencing

Both WT and *aceA* knockout strains were grown to exponential phase (WT OD_600_~0.5, Δ*aceA* OD_600_~0.15) in NaAc-supplemented MSB media. Total RNA was isolated from 10 ml of cells using the RNeasy Mini Kit (Qiagen, USA) according to the manufacturer’s instructions. All procedures for RNA sequencing were conducted by Chunlab (South Korea). The RNA was subjected to a subtractive Hyb-based rRNA removal process using the MICROBExpress Bacterial mRNA Enrichment Kit (Ambion, USA). A library was constructed as described previously^17^. RNA sequencing was performed with two runs of the Illumina Genome Analyzer IIx to generate single-ended 100-bp reads. Quality-filtered reads were aligned to the reference genome sequence using CLC Genomics Workbench 6.5.1 (CLC bio, USA). Mapping was based on a minimal length of 100 bp with an allowance of up to two mismatches. Relative transcript abundance was measured in RPKM. RNA-seq data has been deposited in NCBI under Gene Expression Omnibus (GEO) accession number GSE124640.

### Gene expression analysis by northern blotting

After total RNA was extracted using an RNeasy Kit according to the manufacturer’s instructions, a northern blot analysis was performed as described previously^32^. Briefly, the quantified total RNA samples (2.5 μg) were loaded onto denaturing agarose gels including 0.25 M formaldehyde, separated by gel electrophoresis, and then stained with ethidium bromide to visualize 23S and 16S rRNA. The RNA bands were transferred to nylon membranes (Schleicher and Schuell, Germany) using a TurboBlotter (Schleicher and Schuell, Germany). The membrane was hybridized with a specific ^32^P-labeled probe (Takara, Japan) based on PCR amplification with each primer pair. Autoradiography was performed using an IP plate (Fujifilm, Japan) and a Multiplex Bio-Imaging System (Fujifilm, Japan).

### High-performance liquid chromatography (HPLC) analysis for organic acid detection

DR1 was incubated in 50 mL MSB media containing 1% Hex for 24 h and then centrifuged (1 min, 13,000 × g) to acquire a clear supernatant for the organic acid analysis using UV/visible HPLC (1525, 2707, 2489, 2414, CHM, Waters Co., USA). As previously described^33^, the type of column used was Aminex HPX-87H (Bio-Rad, USA), and the temperature was set to 30 °C. Sulfuric acid (H_2_SO_4_) was used as the solvent, and the flow rate was 0.6 ml/min. For the quantification of intracellular concentration of pyruvate and oxalate, exponentially-grown cells in 100 mL NaAc-added MSB media were collected, and supernatant was discarded. The cell pellet was resuspended in 1 mL of boiling distilled water (DW). After boiling for 5 min, the sample was immediately cooled in ice for 5 min. Cell debris was removed after centrifugation, supernatant was filtered with 0.22 μm filter. The sample was stored at −80 °C before injecting 20 μL into UV/visible HPLC system (Waters Co. USA) equipped with C_18_-column (2.1 × 100 mm, 2.1 μm). The condition of temperature, solvent, and the flow rate was the same as above.

### Construction of *L-ldh* expression strain

The whole region of *L-ldh* gene was amplified using the *L-ldh* forward and reverse expression primers based on DR1 strain (Table S3). The PCR product was digested using *Eco*RI and *Bam*HI restriction enzymes. Fragments were inserted into and ligated with pRK415 vector. The recombinated plasmids were transformed into *E. coli* Top10 strain, and *L-ldh* expression strains were screened onto Luria-Bertani broth agar media containing 20 μg/mL tetracycline. Confirmation was conducted using polymerase chain reaction (PCR) and DNA sequencing.

### Reverse transcription polymerase chain reaction (RT-PCR)

Total RNA was extractedfrom 10 ml of cell cultures using an RNeasy minikit according to the manufacturer’s instructions. Synthesis of cDNA was conducted from 1 μg of RNA with primers for 16S rRNA (b341, b534 primers)^34^ and *L-ldh* gene (Table S3). cDNA was diluted by 10-fold, and amplified under following PCR condition; 95 °C for 5 min, followed by 35 cycles of 15 s at 95 °C, 15 s at 60 °C, and 1 min at 72 °C. Each 5 μl of PCR samples was loaded onto 0.8% agarose gel.

### Susceptibility tests

Wild type and mutant strains were grown overnight in nutrient broth and subsequently diluted 100-fold. The cells reached mid-exponential phase, washed with phosphate buffer saline (PBS, pH 7.4) twice, and approximately 10^6^ cells per ml was resuspended in 1 mL PBS. Serially diluted cells were spotted on nutrient agar containing 0.1–0.4 mM H_2_O_2_.

## Supplementary information

Suppplementary information

## References

[1] Gui L, Sunnarborg A, LaPorte DC (1996). Regulated expression of a repressor protein: FadR activates *iclR*. J Bacteriol..

[2] Fujita Y, Matsuoka H, Hirooka K (2007). Regulation of fatty acid metabolism in bacteria. Mol Microbiol..

[3] Ahn S, Jung J, Jang IA, Madsen EL, Park W (2016). Role of glyoxylate shunt in oxidative stress response. J Biol Chem..

[4] Park C, Park W (2018). Survival and energy producing strategies of alkane degraders under extreme conditions and their biotechnological potential. Front Microbiol..

[5] Jeon JM, Lee HI, Sadowsky MJ, Sugawara M, Chang WS (2015). Characterization of a functional role of the *Bradyrhizobium japonicum* isocitrate lyase in desiccation tolerance. Int J Mol Sci..

[6] Van Acker H, Coenye T (2017). The role of reactive oxygen species in antibiotic-mediated killing of bacteria. Trends Microbiol..

[7] Nandakumar M, Nathan C, Rhee KY (2014). Isocitrate lyase mediates broad antibiotic tolerance in *Mycobacterium tuberculosis*. Nat Commun..

[8] Puckett S (2017). Glyoxylate detoxification is an essential function of malate synthase required for carbon assimilation in *Mycobacterium tuberculosis*. Proc Natl Acad Sci USA.

[9] Ha S, Shin B, Park W (2018). Lack of glyoxylate shunt dysregulates iron homeostasis in *Pseudomonas aeruginosa*. Microbiology..

[10] Meylan S (2017). Carbon sources tune antibiotic susceptibility in *Pseudomonas aeruginosa* via tricarboxylic acid cycle control. Cell Chem Biol..

[11] Shin B, Park W (2017). Antibiotic resistance of pathogenic *Acinetobacter* species and emerging combination therapy. J Microbiol..

[12] Kaur A, Sharma P, Capalash N (2018). Curcumin alleviates persistence of *Acinetobacterbaumannii* against colistin. Sci Rep..

[13] Haruta S, Kanno N (2015). Survivability of microbes in natural environments and their ecological impacts. Microbes Environ..

[14] Kim J, Park C, Imlay JA, Park W (2017). Lineage-specific SoxR-mediated regulation of an endoribonuclease protects non-enteric bacteria from redox-active compounds. J Biol Chem..

[15] Jung J (2015). Molecular mechanisms of enhanced bacterial growth on hexadecane with red clay. Microb Ecol..

[16] Park C, Shin B, Jung J, Lee Y, Park W (2017). Metabolic and stress responses of *Acinetobacter oleivorans* DR1 during long-chain alkane degradation. Microb Biotechnol..

[17] Wang ZX, Brämer CO, Steinbüchel A (2003). The glyoxylate bypass of *Ralstonia eutropha*. FEMSMicrobiol Lett..

[18] Heo A, Jang HJ, Sung JS, Park W (2014). Global transcriptome and physiological responses of *Acinetobacter oleivorans* DR1 exposed to distinct classes of antibiotics. PLoS One..

[19] Roe AJ, O’Byrne C, McLaggan D, Booth IR (2002). Inhibition of *Escherichia coli* growth by acetic acid: a problem with methionine biosynthesis and homocysteine toxicity. Microbiology..

[20] Mandal NC, Chakrabartty PK (1992). Regulation of enzymes of glyoxylate pathway in root-nodule bacteria. J Gen Appl Microbiol..

[21] Heck DE, Shakarjian M, Kim HD, Laskin JD, Vetrano AM (2010). Mechanisms of oxidant generation by catalase. Ann N Y Acad Sci..

[22] Fukaya M (1993). The *aarC* gene responsible for acetic acid assimilation confers acetic acid resistance on *Acetobacter aceti*. J. Ferment. Bioeng..

[23] Koedooder, C., *et al* The role of the glyoxylate shunt in the acclimation to iron limitation in marine heterotrophic bacteria. *Front. Mar. Sci*. In press (2018).

[24] Sharma V, Schwille PO (1992). Oxalate production from glyoxylate by lactate dehydrogenase *in vitro*: inhibition by reduced glutathione, cysteine, cysteamine. Biochem Int..

[25] Kwong WK, Zheng H, Moran NA (2017). Convergent evolution of a modified, acetate-driven TCA cycle in bacteria. Nat Microbiol..

[26] Chen Q, Janssen DB, Witholt B (1995). Growth on octane alters the membrane lipid fatty acids of *Pseudomonas oleovorans* due to the induction of *alkB* and synthesis of octanol. J Bacteriol..

[27] Santos JS, da Silva CA, Balhesteros H, Lourenço RF, Marques MV (2015). CspC regulates the expression of the glyoxylate cycle genes at stationary phase in *Caulobacter*. BMC Genomics..

[28] Hong H, Ko HJ, Choi IG, Park W (2014). Previously undescribed plasmids recovered from activated sludge confer tetracycline resistance and phenotypic changes to *Acinetobacter oleivorans* DR1. Microb Ecol..

[29] Banner MR, Rosalki SB (1967). Glyoxylate as a substrate for lactate dehydrogenase. Nature..

[30] Sugiyama N, Taniguchi N (1997). Evaluation of the role of lactate dehydrogenase in oxalate synthesis. Phytochemistry..

[31] Lorca GL (2007). Glyoxylate and pyruvate are antagonistic effectors of the *Escherichia coli* IclR transcriptional regulator. J Biol Chem..

[32] Kim HJ, Shin B, Lee YS, Park W (2017). Modulation of calcium carbonate precipitation by exopolysaccharide in *Bacillus* sp. JH7. Appl Microbiol Biotechnol..

[33] Villas-Bôas SG, Kesson M, Nielsen J (2005). Biosynthesis of glyoxylate from glycine in *Saccharomyces cerevisiae*. FEMS Yeast Res..

[34] Jung J (2011). Change in gene abundance in the nitrogen biogeochemical cycle with temperature and nitrogen addition in Antarctic soils. Res Microbiol..

[35] Grostern A, Sales CM, Zhuang WQ, Erbilgin O, Alvarez-Cohen L (2015). Glyoxylate metabolism is a key feature of the metabolic degradation of 1,4-dioxane by *Pseudonocardia dioxanivorans* strain CB1190. Appl Environ Microbiol..

[36] Lassalle L (2016). New insights into the mechanism of substrates trafficking in Glyoxylate/Hydroxypyruvate reductases. Sci Rep..

