# Supplementary material for: Alternative fate of glyoxylate during acetate and hexadecane metabolism in Acinetobacter oleivorans DR1
Source: Sci Rep. 2019 Oct 7;9:14402. doi: 10.1038/s41598-019-50852-3 (PMC6779741; doi:10.1038/s41598-019-50852-3)
Supplement: Supplementary file 1 — Suppplementary information [file 41598_2019_50852_MOESM1_ESM.docx]

**Alternative fate of glyoxylate during acetate and hexadecane metabolism in *Acinetobacter oleivorans* DR1**

**Chulwoo Park, Bora Shin, and Woojun Park***

Laboratory of Molecular Environmental Microbiology, Department of Environmental Science and Ecological Engineering, Korea University, Seoul 02841, Republic of Korea

**Running title:** Glyoxylate shunt pathways in *A. oleivorans* DR1

^*^**Corresponding author:** Dr. Woojun Park, Department of Environmental Science and Ecological Engineering, Korea University, Seoul 02841, Republic of Korea

**E-mail:** wpark@korea.ac.kr

**Fax:** +82-2-953-0737

**Phone:** +82-2-3290-3067

**
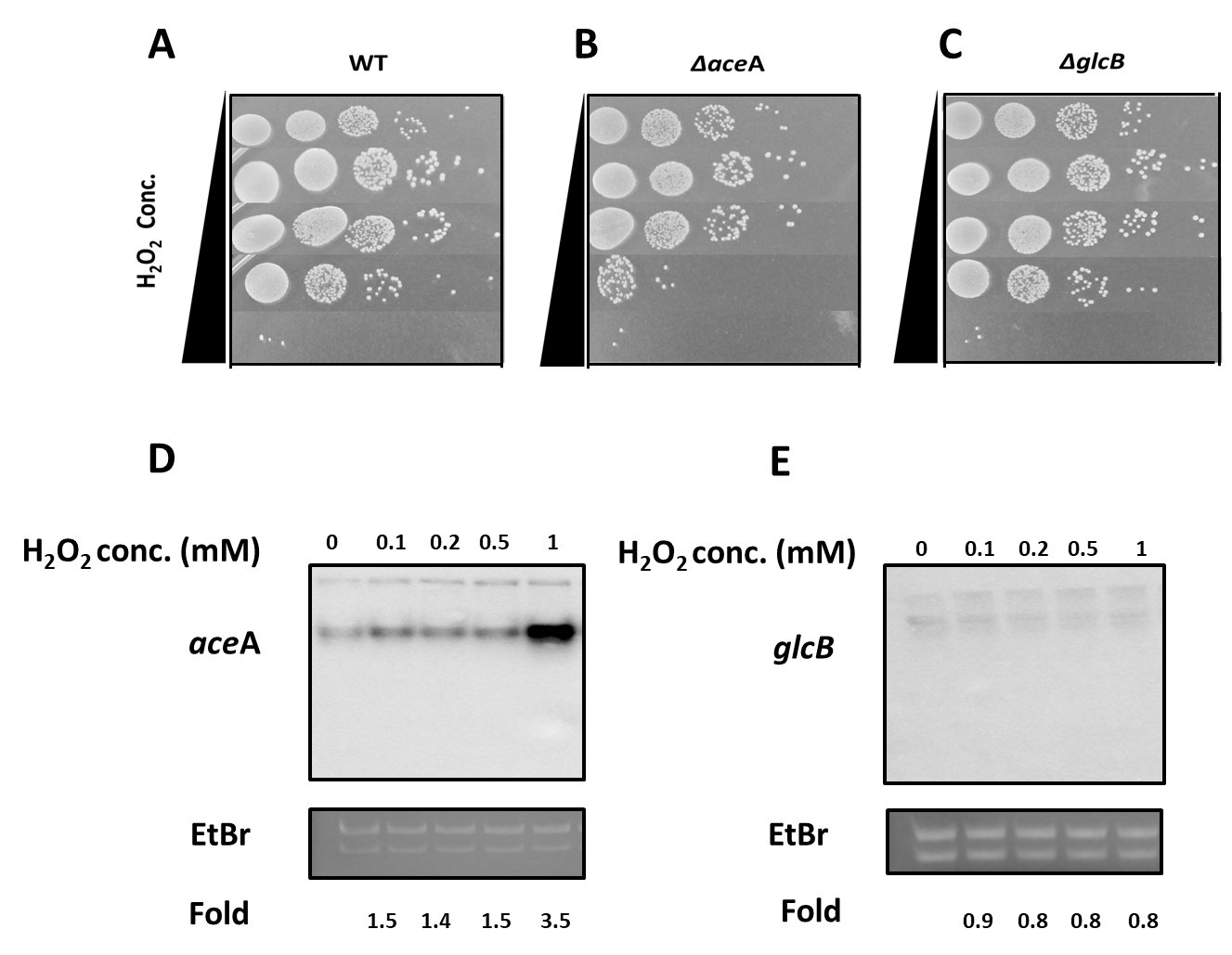
**

**Supplementary Fig. S1.** Survival tests and expression of *aceA* and *glcB* under oxidative stress. Cultures of (A) WT, (B) Δ*aceA*, and (C) Δ*glcB* strains in nutrient broth (NB) were collected and washed. The same number of cells were H_2_O_2_ spotted onto 0 – 0.4 mM H_2_O_2_-containing NB agar media. The concentration of H_2_O_2_ is increasing in the downward direction. (D-E) The expression of (D) *aceA* and (E) *glcB* genes under 0 – 1 mM H_2_O_2_ treatment. Each concentration of H_2_O_2_ was used to treat exponentially grown cells in NB media (OD_600_~0.5) for 15 min..


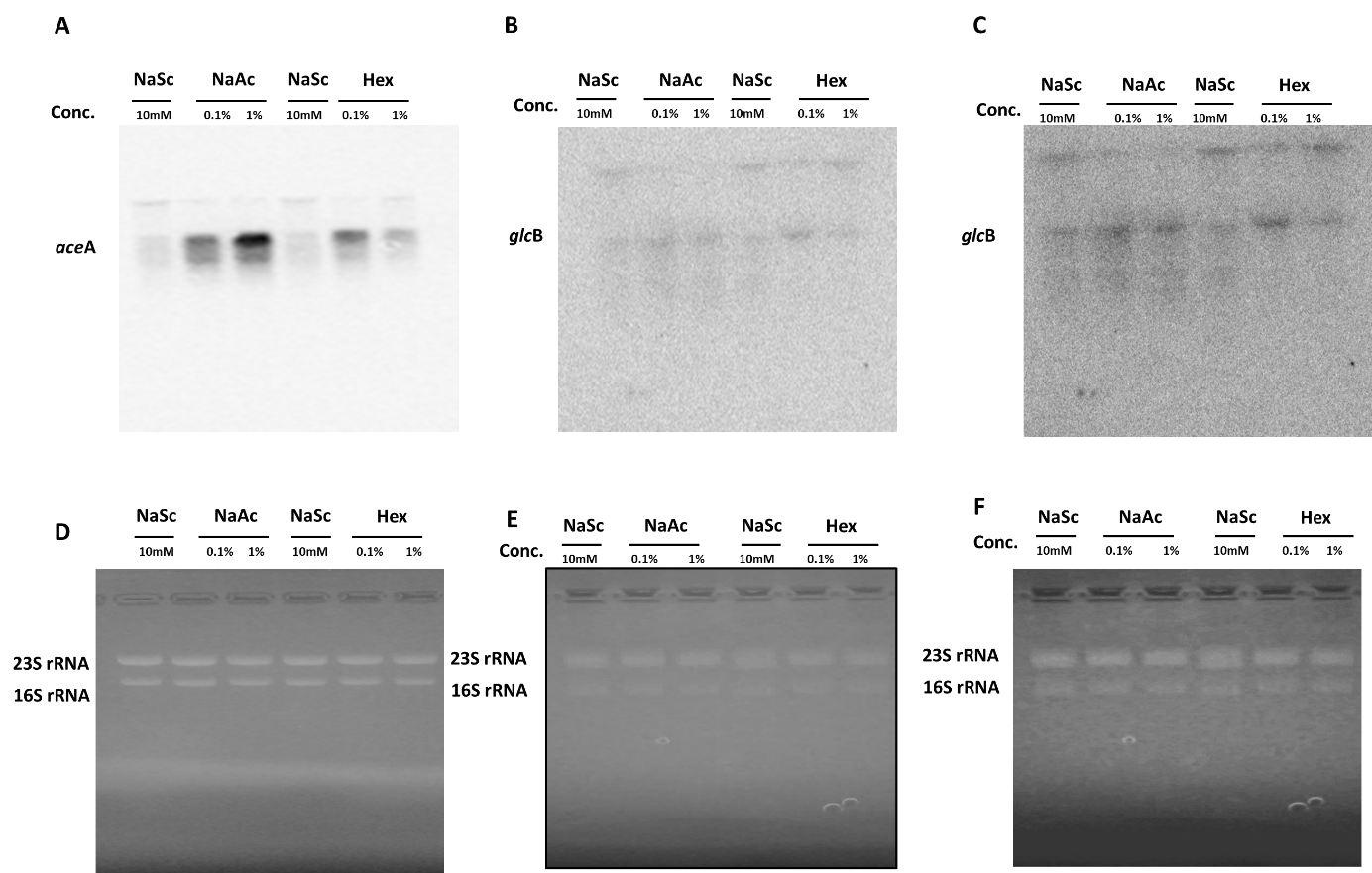


**Supplementary Fig. S2.** The full-length gel and blot images of *aceA* and *glcB*. Orginianl Northern blot images of (A) *aceA*, and (B) *glcB* were shown. (C) To clarify the expression of *glcB*, high-contast was adopted. (D - F) To show the same amounts of each loading sample, 16S rRNA and 23rRNA were visualized using ethidium bromid (EtBr). Original gel images of (D) Penel A and (E) Penel B were demonstrated. (E) The image of Penel E was modified to show clearly.


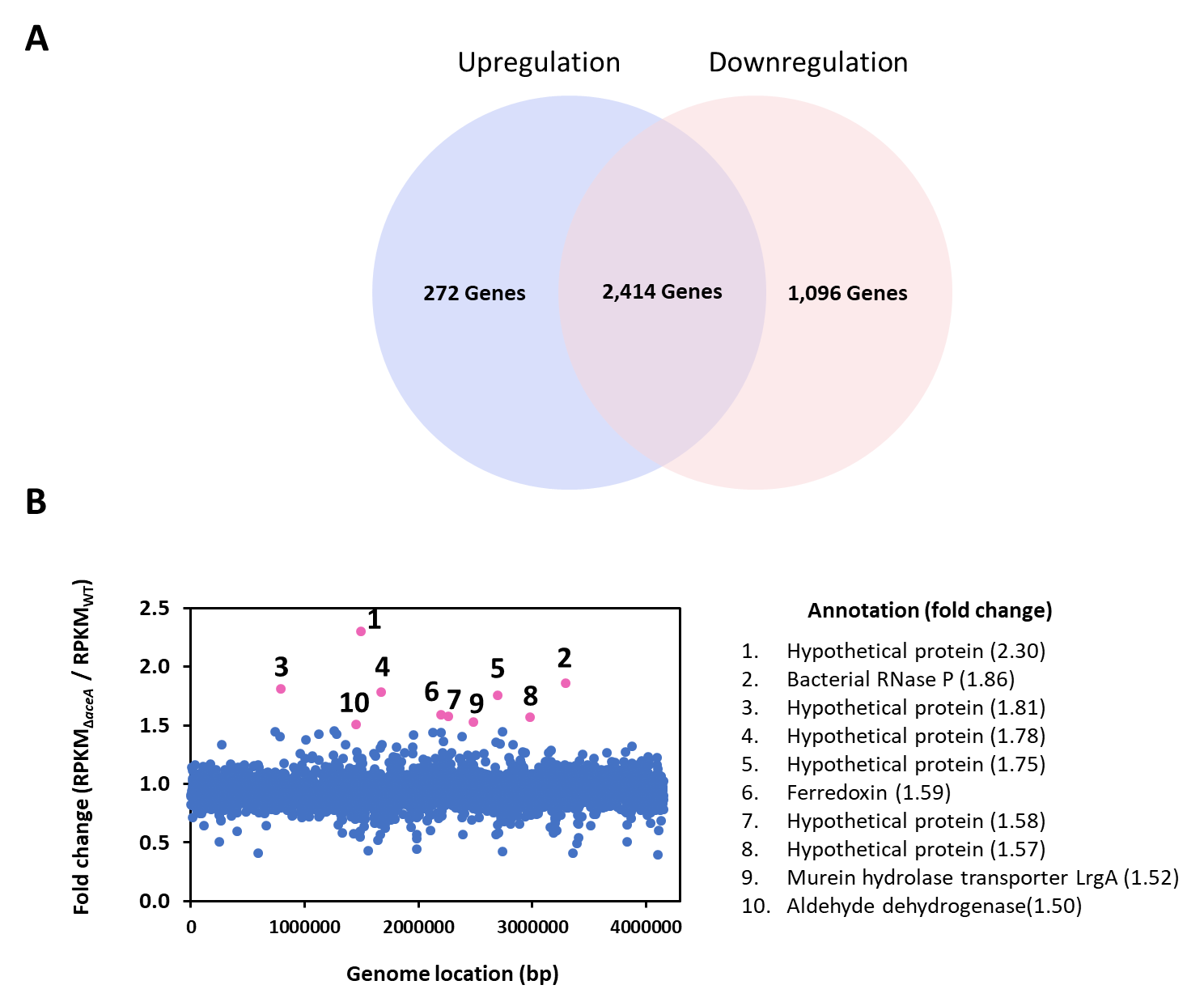


**Supplementary Fig. S3.** Up-, and down-regulated genes in Δ*aceA* mutant compared to that of the wild type strain in the presence of 0.1% NaAc. (A) The number of upregulated and downregulated genes in the absence of ICL; 272 genes were upregulated and 1,096 genes were downregulated groups. No change in expression level was observed for 2,414 genes (0.9 < fold change <1.5). (B) The highest fold change of 10 genes in the Δ*aceA* mutant are displayed. Red dots indicate the genes listed on the right side. X-axis and Y-axis represent the location of the genome and fold change, respectively.


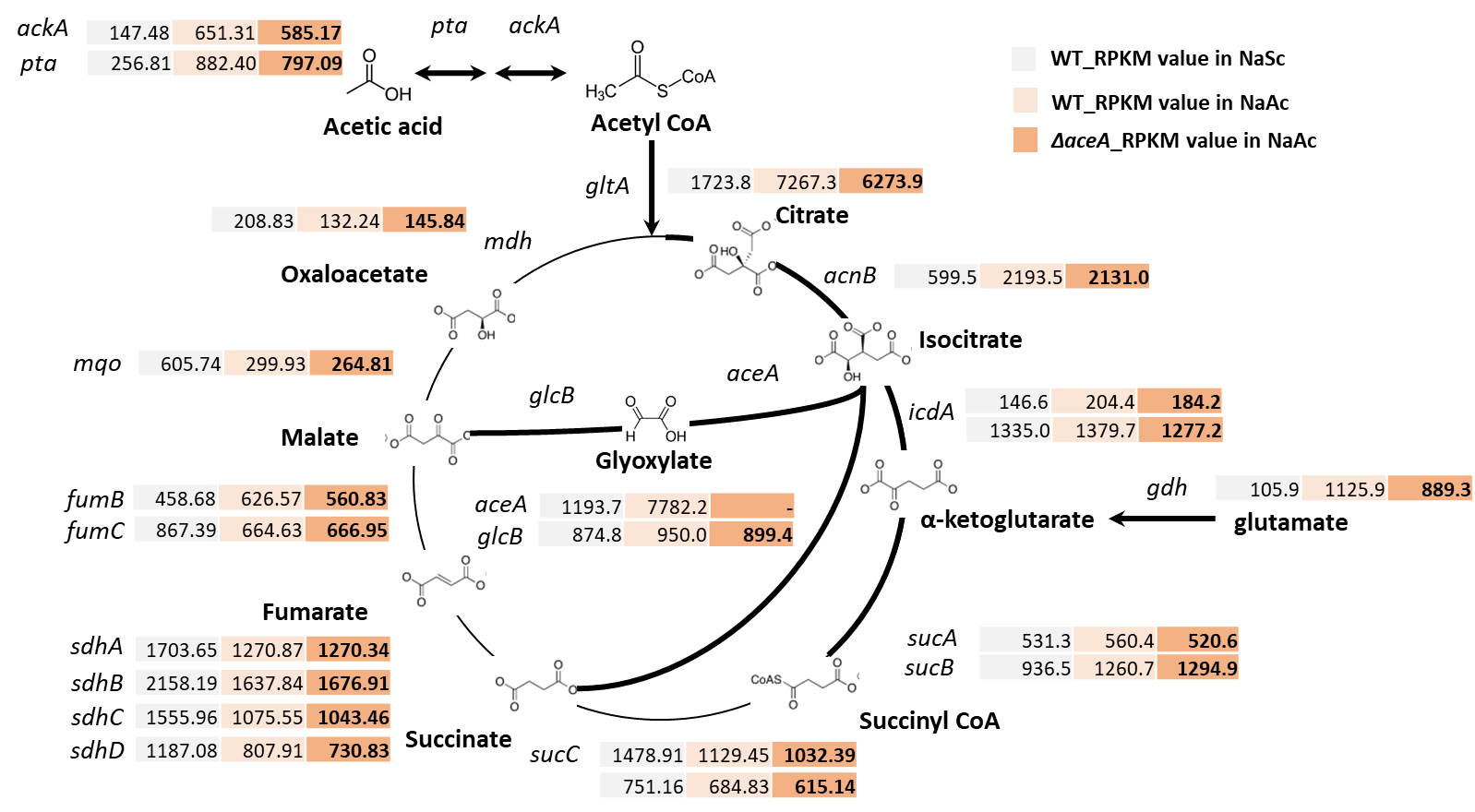


**Supplementary Fig. S4.** Schematic of the expression of genes participating in Pta-AckA, TCA, and GS pathways in wild type in NaSc (gray box) and NaAc (light orange box), and Δ*aceA* mutant on NaAc (dark orange box). The number in boxes indicates reads per kilobase of transcript per million mapped sequence reads (RPKM) values of each sample, and RPKM of Δ*aceA* is highlighted as bold. Thick arrows represent activated pathways in Δ*aceA* mutant during acetate assimilation

**
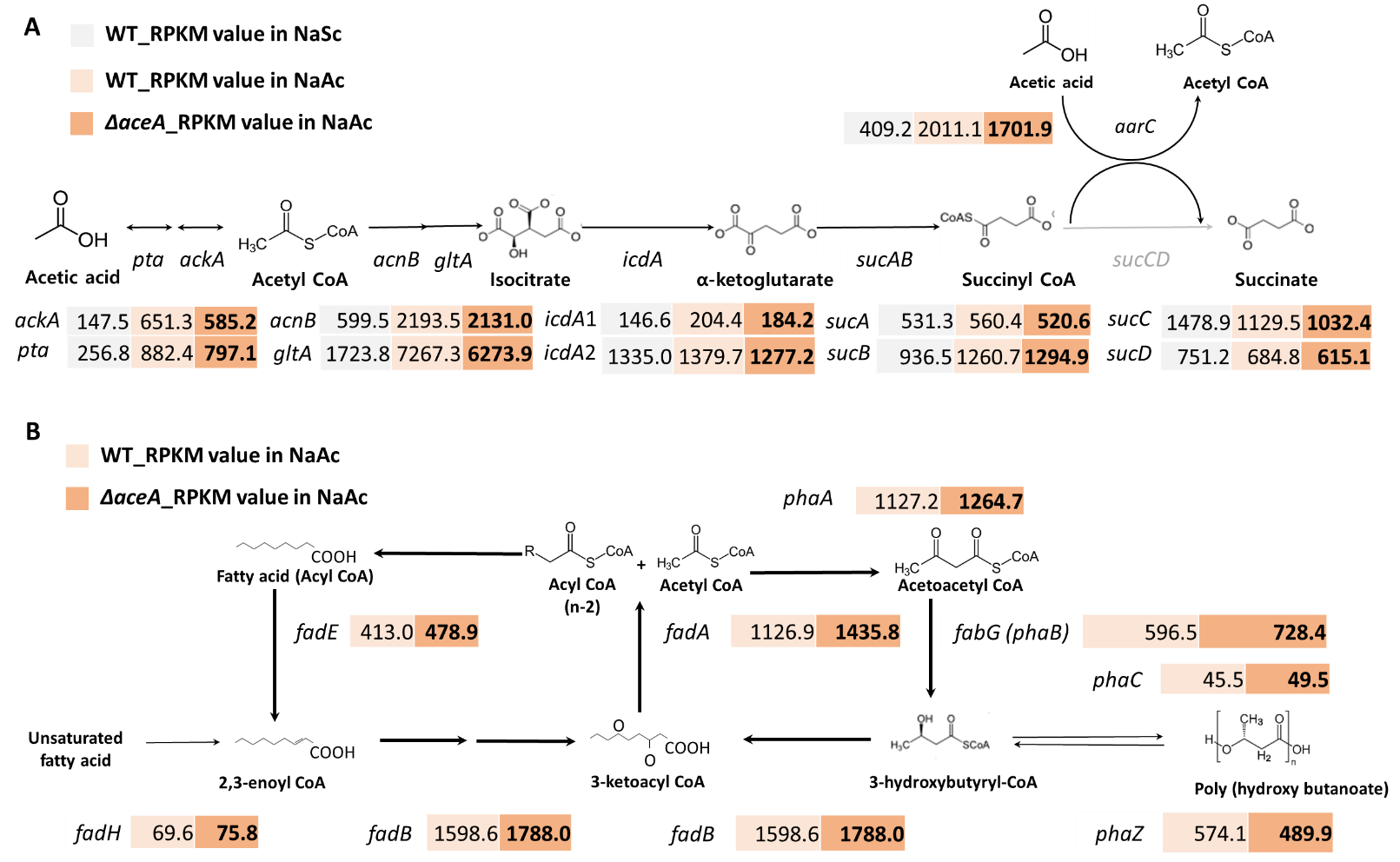
**

**Supplementary Fig. S5.** RNA seq profile of wild type in NaSc (gray box) and NaAc (light orange box), and Δ*aceA* mutant on NaAc (dark orange box). The number in boxes indicates reads per kilobase of transcript per million mapped sequence reads (RPKM) values of each sample, and RPKM of Δ*aceA* is highlighted as bold. (A) Gene expression analysis of isocitrate dehydrogenase (*icdA*), succinyl coenzyme A synthetase (*sucAB*), succinyl coenzyme A ligase (*sucCD*), and succinyl-CoA:acetate CoA transferase (*aarC*). Gray arrow indicates decreased activity in NaAc-supplemented MSB media compared to that in NaSc. (B) Gene expression analysis of genes related to fatty acid oxidation, and PHB-synthesis. Thick arrows represent activated pathways in Δ*aceA* mutant during acetate assimilation.

**Supplementary Table S1.** RNA-seq data information used in this study. Total RNA was obtained from WT and Δ*aceA* mutant cells after exponential growth. RPKM indicates reads per kilobase of transcript per million mapped sequence reads.

|  | **WT** | **Δ*aceA*** |
| --- | --- | --- |
| Total reads | 47,584,896 | 42,242,295 |
| Total mapped reads (%) | 97.6 | 97.8 |
| Average RPKM | 329.0 | 340.6 |
| Median RPKM | 69.2 | 64.9 |

**Supplementary Table S2.** Transcriptomic analysis data of parental strain and Δ*aceA* mutant under growth in media containing 0.1% NaAc.

| **Locus tag** | **Gene symbol** | | **RPKM** | | | **Annotation** | | **Fold change** | | |
| --- | --- | --- | --- | --- | --- | --- | --- | --- | --- | --- |
|  |  |  | **WT** | | **Δ*aceA*** |  |  |  |  |  |
| **Respiration and ATP synthesis** | | | | | | | | | | |
| AOLE_RS06455 | *cyoB* | | 5768.4 | | 6077.6 | | Cytochrome O ubiquinol oxidase |  | | 1.1 |
| AOLE_RS15870 | *nuoI* | | 1291.6 | | 1490.8 | | NADH-quinone oxidoreductase subunit I |  | | 1.2 |
| AOLE_RS18620 | *atpE* | | 7197.9 | | 7790.9 | | F_0_F_1_ ATP synthase subunit epsilon |  | | 1.1 |
| AOLE_RS18660 |  | | 166.5 | | 199.05 | | ATP synthase subunit |  | | 1.2 |
| AOLE_RS17565 | *etfD* | | 1239.5 | | 1379.7 | | Probable electron transfer flavoprotein-ubiquinone oxidoreductase |  | | 1.1 |
| AOLE_RS18625 | *atpB* | | 8789.1 | | 9008.8 | | ATP synthase subunit beta |  | | 1.0 |
| AOLE_RS18630 | *atpC* | | 6605.8 | | 6625.0 | | F_0_F_1_ ATP synthase subunit gamma |  | | 1.0 |
| AOLE_RS18635 | *atpA1* | | 7105.5 | | 7131.0 | | ATP synthase subunit alpha 1 |  | | 1.0 |
| **Motility** | | | | | | | | | | |
| AOLE_RS00535 | *cheY* | | 193.8 | 208.6 | | | Chemotaxis protein |  | 1.1 | |
| AOLE_RS03250 | *pilT* | | 218.8 | 240.5 | | | Twitching motility protein |  | 1.1 | |
| AOLE_RS16950 | *pilW* | | 337.4 | 379.0 | | | Pilus assembly protein |  | 1.1 | |
| AOLE_RS17815 | *pilF* | | 59.8 | 65.5 | | | Type IV pilus assembly protein |  | 1.1 | |
| AOLE_RS18280 | *pilS* | | 82.3 | 88.1 | | | Sensor protein |  | 1.1 | |
| **Cation transport/regulator and metal-associated proteins** | | | | | | | | | | |
| AOLE_RS01615 | *bfr* | 175.8 | | 205.9 | | | Regulatory or redox protein |  | 1.2 | |
| AOLE_RS08240 | *grx* | 633.0 | | 675.0 | | | Glutaredoxin |  | 1.1 | |
| AOLE_RS10635 | *iscA* | 652.1 | | 762.5 | | | FeS assembly scaffold |  | 1.2 | |
| AOLE_RS12970 | *fdx* | 521.6 | | 561.7 | | | Ferredoxin |  | 1.1 | |
| AOLE_RS13285 | *merR* | 199.7 | | 224.0 | | | Mercuric resistance operon  regulatory protein |  | 1.1 | |
| AOLE_RS13295 | *sufA* | 1887.9 | | 2150.7 | | | Heavy-metal-associated domain protein |  | 1.1 | |
| AOLE_RS14420 | *nfuA* | 163.8 | | 177.9 | | | Fe/S biogenesis protein |  | 1.1 | |
| AOLE_RS14775 | *fec* | 90.6 | | 116.5 | | | Iron ABC transporter permease |  | 1.3 | |
| AOLE_RS15210 | *furR* | 1223.5 | | 1308.4 | | | Fur family transcriptional regulator |  | 1.1 | |
| AOLE_RS15440 | *fdx* | 350.8 | | 373.3 | | | Ferredoxin |  | 1.1 | |
| AOLE_RS16205 |  | 73.3 | | 82.5 | | | Cation transporter |  | 1.1 | |
| AOLE_RS17545 | *hemH* | 478.9 | | 509.3 | | | Ferrochelatase |  | 1.1 | |
| AOLE_RS18240 | *feoB* | 441.0 | | 498.1 | | | Iron transporter |  | 1.1 | |
| AOLE_RS18245 |  | 806.4 | | 947.6 | | | Iron transporter |  | 1.2 | |
| **Stress response** | | | | | | | | | | |
| AOLE_RS02000 |  | 276.2 | | 301.7 | | | ATP-dependent RNA helicase-like  protein DB10 |  | 1.1 | |
| AOLE_RS04700 | *recO* | 127.7 | | 139.5 | | | DNA repair protein |  | 1.1 | |
| AOLE_RS05875 | *cspA* | 1376.4 | | 2001.3 | | | Cold-shock protein |  | 1.5 | |
| AOLE_RS06005 | *cspE* | 1694.7 | | 2413.4 | | | Cold-shock protein |  | 1.4 | |
| AOLE_RS11430 | *ahpF1* | 4145.2 | | 4821.0 | | | Alkyl hydroperoxide reductase |  | 1.2 | |
| AOLE_RS13385 | *hrpA* | 110.1 | | 119.0 | | | ATP-dependent RNA helicase |  | 1.1 | |
| AOLE_RS13420 | *ahpF2* | 4728.3 | | 5261.8 | | | Alkyl hydroperoxide reductase |  | 1.1 | |
| AOLE_RS15835 | *cspG* | 181.2 | | 193.6 | | | Cold-shock protein |  | 1.1 | |
| AOLE_RS17405 | *katG* | 2269.7 | | 2559.6 | | | Catalase-peroxidase |  | 1.1 | |
| AOLE_RS17865 | *ruvX* | 756.2 | | 899.6 | | | Holliday junction DNA helicase |  | 1.2 | |
| AOLE_RS17910 | *uvrC* | 153.6 | | 164.7 | | | UvrABC system protein C |  | 1.1 | |

**Supplementary Table S3.** List of candidate glyoxylate-detoxifier homologues in *A*. *oleivorans* DR1 using blastP.

| **Query**  **(Gene symbol,**  **Accession no.)** | **Identifier** | **Cover** | **E-value** | **Similarity** | **Reference** |
| --- | --- | --- | --- | --- | --- |
| hydroxypyruvate/  glyoxylate reductase  (*yiaE*, AE000432) | AOLE_RS05940 | 96% | 2.00E-93 | 46% | Nunez et al (2001) |
| glyoxylate carboligase  (*gcl*, CQR80105) | AOLE_RS16745 | 92% | 1.00E-86 | 32% | Cusa et al (1999) |
| alanine glyoxylate transaminase  (*agt*, BAB40321) | AOLE_RS18375 | 89% | 5.00E-34 | 28% | Sakuraba et al (2004) |
| alanine glyoxylate transaminase  (*agt*, BAB40321) | AOLE_RS07330 | 80% | 4.00E-32 | 28% | Sakuraba et al (2005) |
| L-lactate dehydrogenase  (*L-ldh*, NP_418062) | AOLE_RS19080 | 96% | 0 | 87% | - |
| D-lactate **d**ehydrogenase  (*D-ldh*, AML01311) | AOLE_RS19075 | 98% | 0 | 56% | - |
| tartronic semialdehyde reductase  (*glxR*, AKK16832) | AOLE_RS09255 | 97% | 5.00E-73 | 45% | Cusaet al (1999) |

**Supplementary Table S3.** Primers used for knock-out and Northern blot experiments in this study.

| **Gene name** | **Locus tag** | **Product** | **Primer  Forward (5'-3') Reverse (3'-5')** |
| --- | --- | --- | --- |
| Knock-out | | | |
| *glcB* | AOLE_RS10780 | malate synthase G | CGCCCCGGGGAAGCTGCACCAGTTGTTCG  CGCGGTACCCTGGTGCAAAGGGAGGAGTT |
| *L-ldh* | AOLE_RS19080 | L-lactate dehydrogenase | CGCGGTACCGGGCCATCCCAAAATTCACG  CGCGAATTCGCCCTATGTGGTTCCAGCTT |
| *D-ldh* | AOLE_RS19075 | D-lactate dehydrogenase | CGCGGTACCTTTGGGAACATTGGCTGTGC CGCGAATTCAAGCAGGCATCGGACTATCG |
| Northern blot | | | |
| *aceA* | AOLE_RS14285 | Isocitrate lyase | CTCAGAAGGCGCTGACTTGA  CGTTTGGAGTCGCAGTTTCG |
| *glcB* | AOLE_RS10780 | Malate synthase G | CAGGGCCGCAGTTAGTTGTA  CTCAGAAGGCGCTGACTTGA |
| *L-ldh* expression | | | |
| *L-ldh* | AOLE_RS19080 | L-lactate dehydrogenase | CGCCTCGAGAGGCTGTGCTTATTGCCTCA  CGCGAATTCAGGCTGTGCTTATTGCCTCA |
| RT-PCR | | | |
| *L-ldh* | AOLE_RS19080 | L-lactate dehydrogenase | AGGCTGTGCTTATTGCCTCA  AAGACGAAGCTCGCCAGAAA |
